# Patients' preferences for secondary prevention following a coronary event

**DOI:** 10.1016/j.pmedr.2024.102681

**Published:** 2024-03-08

**Authors:** Tinka J. van Trier, Harald T. Jørstad, Wilma J.M. Scholte op Reimer, Madoka Sunamura, Nienke ter Hoeve, G. Aernout Somsen, Ron J.G. Peters, Marjolein Snaterse

**Affiliations:** a Department of Cardiology, Amsterdam University Medical Centers, University of Amsterdam, Amsterdam, The Netherlands; b Research Group Chronic Diseases, HU University of Applied Sciences Utrecht, Utrecht, The Netherlands; c Capri Cardiac Rehabilitation Rotterdam, Rotterdam, The Netherlands; d Franciscus Gasthuis & Vlietland Hospital, Rotterdam, The Netherlands; e Department of Rehabilitation Medicine, Erasmus MC Medical Center, Rotterdam, The Netherlands; f Cardiology Centers of the Netherlands, Amsterdam, The Netherlands

**Keywords:** Patient preferences, Risk factors, Lifestyle, Cardiovascular disease, Rehabilitation

## Abstract

**Objective:**

Despite clear evidence on the effectiveness of secondary prevention, patients with coronary artery disease frequently fail to reach guideline-based risk factor targets. Integrating patients’ preferences into treatment decisions has been recommended to reduce this gap. However, this requires knowledge about patient treatment preferences. Therefore, through a survey study, we aimed to explore which risk factors patients self-perceived, prioritised for improvement, and needed support with after a recent hospitalisation for coronary heart disease.

**Methods:**

A digital questionnaire was presented to patients > 18 years recently discharged (≤3 months) from an acute coronary care unit in the Netherlands (Europe). Patients could select from eight cardiovascular risk factors that they (1) self-perceived, (2) prioritised for improvement, and (3) needed support to improve. Patients’ perceived risk factors were compared to those documented in the medical records.

**Results:**

Respondents (N = 254, 26 % women), mean age 64 (SD 10) years, identified ‘physical inactivity’ more frequently than their medical records (140 patients vs. 91 records, p < 0.001), while three other risk factors were reported with equal and four with lower frequency. ‘Physical inactivity’, ‘overweight’ and ‘stress’ were most frequently prioritised for improvement (82 %, 88 % and 78 %) and professional support (64 %, 50 % and 58 %), with 87 % preferring lifestyle optimisation if this would reduce drug use.

**Conclusions:**

Patients with a recent coronary event show significant disparities in identifying risk factors compared to their medical records. They tend to prefer improving lifestyle- over drug-modifiable risk factors, particularly physical inactivity, overweight and stress, and indicate the need for support in improving these factors.

## Highlights

• Patients’ risk factor perception markedly differs from their medical records.
• Patients rate ‘physical inactivity’ as most applicable and important to improve.
• Patients prefer improving lifestyle above drug therapy intensification.
• Integrating patient preferences may facilitate personalised treatment decisions.

## 1 Introduction

To prevent future cardiovascular events, prevention guidelines recommend to optimise pharmacological treatment and lifestyle (Visseren et al., 2021). However, the majority of coronary patients fail to meet guideline-based targets (Kotseva et al., 2019, Ray et al., 2020, van Trier et al., 2023) due to various reasons, including non-adherence to medication, underutilisation of rehabilitation and lifestyle programs, and failure to maintain lifestyle changes (Naderi et al., 2012, Tijssen et al., 2022).

To bridge this gap, it is recommended to improve patient-physician communication (Sarkar et al., 2011), patients’ self-perception of risk factors (Thakkar et al., 2016, Soroush et al., 2017), and integrate patient preferences into personalised treatment decisions (Pedretti et al., 2022, Franklin et al., 2020, Elwyn et al., 2012). Hence, understanding patient preferences after acute hospitalisation – which constitutes a teachable moment for lifestyle change (Tofler et al., 2015) – is important for implementing this recommendation.

By eliciting patient preferences, healthcare providers can better understand the patients perspective and tailor preventive treatments to the individual patients need. This approach may result in improved compliance and outcomes (Pedretti et al., 2022, Franklin et al., 2020, Shay and Lafata, 2015). Therefore, the aim of this survey study was to determine patients’ risk factor perception and treatment preferences after a recent hospitalisation for coronary heart disease, to improve the implementation of guideline-based preventive treatments.

## 2 Methods

### 2.1 Study design

A cross-sectional survey study was conducted using a digital, self-administered questionnaire developed from relevant literature on patient risk perception, informed decision-making, and patient preferences (Thakkar et al., 2016, Soroush et al., 2017, Franklin et al., 2020, Elwyn et al., 2012, Shay and Lafata, 2015). Items were formulated by cardiovascular healthcare professionals to ensure content validity, and reviewed by the national patient association. The full questionnaire is included in the original Dutch (Appendix 1) and a translated English version (Appendix 2). In this analysis, we focused on a selection of three topics answered through questions with nine multiple-choice options (‘smoking’, ‘overweight’, ‘physical inactivity’, ‘stress’, ‘depression’, ‘high blood pressure’, ‘high cholesterol’, ‘high blood sugar’ or ‘none’):

1. ‘What applies to you?’ (to compare self-perceived risk factors with the medical record, based on question 1);
2. ‘Which of these (maximum three) risk factors do you consider most important to improve?’ (to examine patients’ priorities, based on questions 2, 5 and 8) and
3. ’I would like to receive help in improving…’, (to investigate patients need for support, based on question 11).

Medical definitions of the risk factors presented as multiple choice options were deliberately omitted to explore patients’ interpretations irrespective of their alignment with established medical classifications. In addition, we asked whether patients preferred lifestyle change versus more medication (based on questions 15 and 16). The Medical Ethics Assessment Committee (METC) of the Amsterdam University Medical Center granted an ethical exemption for the study, as it did not fall under the scope of the Medical Research Involving Human Subjects Act (WMO).

### 2.2 Patients and data collection

Patients > 18 years discharged ≤ 3 months after hospitalisation for an acute coronary syndrome or revascularisation procedure were included before starting cardiac rehabilitation. Exclusion criteria were insufficient Dutch language proficiency or no Participants received the questionnaire by e-mail and provided written informed consent to access their medical records. Recorded risk factors were defined according to European guidelines (Visseren et al., 2021) (Appendix, table A) and collected from hospital discharge letters and cardiac rehabilitation records.

### 2.3 Statistical analysis

Baseline characteristics are presented as proportions (categorical), mean with standard deviations (SD) (normal distributed continuous), or median and interquartile ranges (IQR). Patients who did not complete the questionnaire or completed it > 3 months after hospitalisation were excluded from the analysis. Risk factors identified by patients and registered in the medical record were compared as paired data at group level using McNemar test. Disparity between patient response and medical record on the presence of a risk factor was defined at the individual level, i.e. the percentage of cases where patients’ and medical reports did not both indicate the presence or absence of a risk factor. R (R studio, version 4.1.3) was used to perform all statistical analyses.

## 3 Results

A total of 481 patients met inclusion criteria, of which 65 had no e-mail access and 12 were not proficient in the Dutch language. Of 404 eligible patients, 306 completed the survey (from February 2020 until May 2021), yielding a response rate of 76 %. After excluding 9 late respondents and 43 patients without consent (details in Appendix, table B), the final cohort included 254 patients. They had a median age of 64 (SD 10) years at a median of 38 (IQR 25–52) days after discharge, with 26 % being women, 78 % native Dutch and 40 % having a high educational level. Medical history included diabetes mellitus in 20 %, a prior myocardial infarction or revascularisation in 22 %, and stroke in 6 %. During hospitalisation, 93 % underwent revascularisation, mainly due toacute coronary syndrome (77 %), with the remaining being elective. Notably, for 72 %, this hospitalisation was their first cardiovascular event..

### 3.1 Risk factor perception

**Fig. 1:**
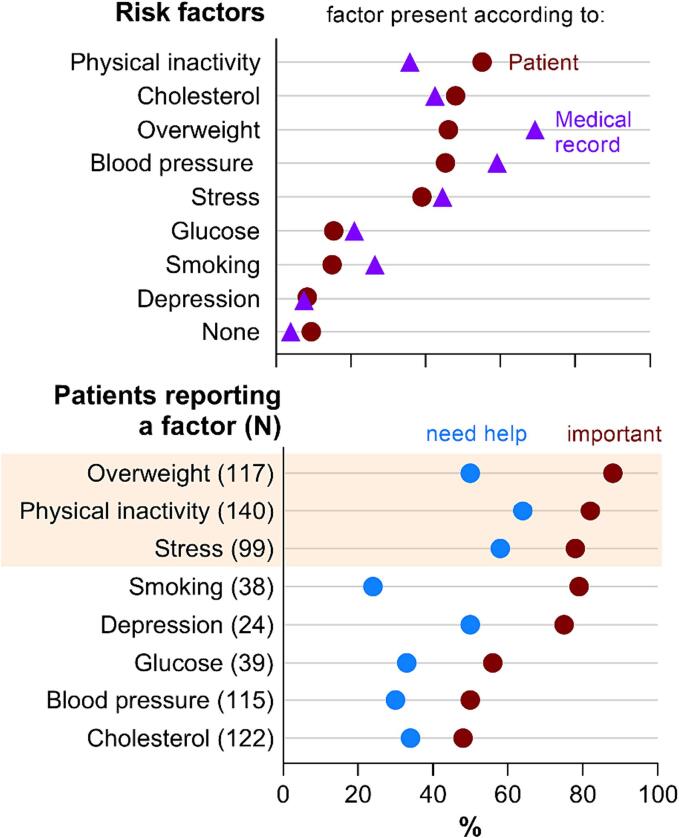
**Cardiovascular risk factors reported by 254 Dutch adult patients within three months after coronary event.** Upper panel: applicable according to patients (red dots) compared to their medical records (triangles). Lower panel: percentage of patients that reported the risk factor as important to improve (red dots) and for which professional help is desired (blue dots). The number of patients who perceived a risk factor is reported after the y-axis label and determines the denominator. Top answers given (stress, overweight and physical inactivity) are highlighted by a semi-transparent rectangle.

In total, 91 % of patients identified having ≥ 1 risk factor, with a median of 3 (IQR 2–4) risk factors per patient. Patients most frequently identified ‘physical inactivity’ (55 %), ‘high cholesterol’ (48 %) and ‘overweight’ (46 %) as applicable risk factors.(Fig. 1, upper panel) ‘Physical inactivity’ (n = 140) and having ‘no risk factors’ (n = 24) were more frequently identified by patients than by medical records (n = 91, p < 0.001 and n = 10, p = 0.01, respectively). Conversely, patients less frequently identified ‘high blood pressure’ (115 vs. 150 medical records, p < 0.001), ‘overweight’ (117 vs. 176, p < 0.001), ‘smoking’ (38 vs. 67, p < 0.001) and ‘high blood sugar’ (39 vs. 53, p = 0001). At the individual level, the greatest patient-medical disparities were for the presence or absence of ‘high cholesterol’ (in 39 % of cases, at group level patients = record), ‘physical inactivity’ (34 % disparity, group level patients > record) and ‘high blood pressure’ (34 % disparity, group level record > patients), while responses were most similar for ‘no risk factors’ (10 % disparity), ‘depression’ (9 %) and ‘high blood sugar’ (7 %) (details in Table 1).

**Table 1:** **Comparison between cardiovascular risk factors reported by 254 Dutch adult patients within three months after coronary event and their medical record at group- and individual-level.** P-value < 0.05 indicates significant group level difference, calculated with McNemar test. Individual-level disparity was defined as the percentage of cases where patients’ and medical reports did not both indicate the presence or absence of a risk factor.

	**According to**		**Group level difference** **p-value**	**Individual-level** **disparity**
**Risk factors**	**Patient**	**Medical record**		
	**n (%)**	**n (%)**		
**Cholesterol**	122 (48 %)	108 (43 %)	0.19	39 %
**Physical inactivity**	140 (55 %)	91 (36 %)	<0.001	34 %
**Blood pressure**	115 (45 %)	150 (59 %)	<0.001	34 %
**Overweight**	117 (46 %)	176 (69 %)	<0.001	26 %
**Stress**	99 (39 %)	113 (44 %)	0.10	25 %
**Smoking**	38 (15 %)	67 (26 %)	<0.001	12 %
**None**	24 (9 %)	10 (4 %)	0.01	10 %
**Depression**	24 (9 %)	19 (7 %)	0.40	9 %
**Blood sugar**	39 (15 %)	53 (21 %)	0.001	7 %

### 3.2 Patients’ preferences

Of 230 patients who reported having risk factor(s), 94 % prioritised ≥ 1 risk factor for improvement, most frequently ‘overweight’ (88 %), ‘physical inactivity’ (82 %), ‘smoking’ (79 %) and ‘stress’ (78 %)(Fig. 1, lower panel). In contrast, the least prioritised were ‘high cholesterol’ (48 %) and ‘high blood pressure’ (50 %). Of note, when asked whether patients were motivated to optimise their lifestyle if this would reduce the number of drugs, 87 % replied with ‘yes’, with only 4 % indicating a preference for more medication instead of lifestyle change.

In total, 82 % of patients indicated a need for professional support to improve ≥ 1 of their perceived risk factors, most frequently for ‘physical inactivity’ (64 %), ‘stress’ (58 %), ‘depression’ (50 %) and ‘overweight’ (50 %). Conversely, patients least often preferred help for ‘smoking’ (24 %), ‘high blood pressure’ (30 %) and ‘high blood sugar’ (33 %) (Fig. 1, lower panel).

## 4 Discussion and conclusion

This survey study shows a marked difference between coronary patients’ and physicians’ perceptions of risk factors, with patients highlighting physical inactivity, but underestimating blood pressure, overweight, smoking and glucose. Patients prioritised physical inactivity, overweight and stress for improvement and support. Notably, 94 % acknowledged the importance of improving their risk factors, with a clear preference for lifestyle-related risk factors, and 82 % indicated the need for professional support in such lifestyle changes. Therefore, assessing and incorporating patient preferences at the outset of prevention programs may be a useful starting-point to tailor secondary prevention to individual needs.

Our finding that patients’ risk factor perception differs from healthcare professionals aligns with previous research, which also indicates that cardiac rehabilitation patients document less applicable risk factors and attribute greater importance to ‘physical inactivity’ than healthcare professionals (Soroush et al., 2017, Reges et al., 2011, Navar et al., 2021, Ter Hoeve et al., 2022, Liu et al., 2023). Furthermore, our study demonstrates that although patients and healthcare professionals report ‘high cholesterol’ with equal frequency at the group level, there was a notable mismatch (39 %) at individual-level identification. This finding highlights significant disparities, possibly stemming from varying comprehension, perspectives and definitions of cardiovascular risk factors between patients and physicians (Sarkar et al., 2011, Soroush et al., 2017, Reges et al., 2011). Such differences could negatively influence patient-physician communication, treatment-decisions and adherence.

Our findings emphasise the need to align patients’ and doctors’ perception of coronary heart disease risk. This alignment can be achieved through patient health education and by physicians exploring and respecting individual patients’ preferences. In this context, particular attention is required to elicit and incorporate patients' preferences during consultations, to foster patient autonomy and self-determination (Shay and Lafata, 2015). To facilitate effective patient-physician communication, it is vital to circumvent the use of medical jargon and pre-emptive assumptions on patients’ interpretations of risk factors (Pedretti et al., 2022). Ultimately, the potential benefits of appropriate individual risk factor management are substantial, both in life years saved, in quality of life and in the prevention of costly recurrent events (van Trier et al., 2023).

Limitations included the pre-selection of nine multiple choice risk factors considered medically important, the use of potentially incomplete medical records for risk factor registration (Moghei et al., 2019) and selection bias due to the inclusion of patients referred to cardiac rehabilitation. Finally, the COVID-19 pandemic, which coincided with this study, may have influenced responses through changed lifestyle habits and awareness.

## 5 Conclusion

Patients with a recent coronary event show significant disparities in identifying risk factors compared to their medical records. They tend to prefer improving lifestyle- over drug-modifiable risk factors, particularly physical inactivity, overweight and stress, and indicate the need for support in improving these factors. Integrating patient preferences into treatment decisions necessitates effective communication and could potentially bridge the gap between clinical practice and achieving guideline-mandated treatment- and lifestyle targets after an acute coronary event.

## Funding

The current study was supported by the 10.13039/501100003246Dutch Research Council (SIA/NWO), grant number HBOPD.2018.02.035, and by an unrestricted grant from AMGEN.

## CRediT authorship contribution statement

**Tinka J. van Trier:** Writing – original draft, Visualization, Project administration, Methodology, Investigation, Formal analysis, Data curation, Conceptualization. **Harald T. Jørstad:** Writing – review & editing, Visualization, Supervision, Methodology, Conceptualization. **Wilma J.M. Scholte op Reimer:** Visualization, Supervision, Methodology, Conceptualization. **Madoka Sunamura:** Writing – review & editing, Data curation. **Nienke ter Hoeve:** Writing – review & editing, Data curation. **G. Aernout Somsen:** Writing – review & editing, Data curation. **Ron J.G. Peters:** Writing – review & editing, Supervision, Methodology, Conceptualization. **Marjolein Snaterse:** Writing – review & editing, Visualization, Supervision, Methodology, Funding acquisition, Data curation, Conceptualization.

## Declaration of competing interest

The authors declare the following financial interests/personal relationships which may be considered as potential competing interests: Marjolein Snaterse reports financial support was provided by Dutch Research Council (SIA/NWO), grant number 23 HBOPD.2018.02.035. Harald Jorstad reports financial support was provided by an unrestricted grant from AMGEN. The other authors (Tinka J. van Trier, Wilma JM. Scholte op Reimer, Madoka Sunamura, Nienke ter Hoeve, G. Aernout Somsen and Ron J.G. Peters) of the manuscript ‘Patients' Preferences for Secondary Prevention Following a Coronary Event’ have nothing to declare; none of them have competing financial interests or personal relationships that could have appeared to influence the work reported in this paper.

## Data Availability

Data will be made available on request.
