# Supplementary material for: Patients' preferences for secondary prevention following a coronary event
Source: Prev Med Rep. 2024 Mar 8;40:102681. doi: 10.1016/j.pmedr.2024.102681 (PMC10940170; doi:10.1016/j.pmedr.2024.102681)
Supplement: Supplementary data 1 [file mmc1.docx]

**APPENDICES DIGITAL CONTENT**

**Patients' preferences for secondary prevention following a coronary event -** van Trier et al.

**Table A Definitions of risk factors according to the medical record.**

**Table B Patient characteristics stratified by reasons not being included in final analysis.**

**Table C Definitions of educational level and ethnical origin.**

| **Risk factor** | **Definition in medical record** |
| --- | --- |
| Smoking | Current smoking of tobacco in any form |
| Overweight | Body Mass Index >25 kg/m^2^ |
| Physical inactivity | <150 minutes of physical activity per week |
| Stress | The word ‘stress’ mentioned in relation to work, health or personal life |
| Depression | Depression in medical history |
| High blood pressure | 1. Hypertension in medical history, or 2. Systolic blood pressure >140 mmHg after acute hospitalisation |
| High cholesterol | 1. Hypercholesterolemia in medical history, or 2. Low-density lipid lipoprotein cholesterol >1.8 mmol/L |
| High glucose | 1. Diabetes mellitus in medical history, or 2. Fasting glucose >7 mmol/L or non-fasting glucose >11 mmol/L |

**Appendix table A. Definitions of risk factors according to the medical record.** Definitions were based on the 2016 European Guidelines on cardiovascular disease prevention in clinical practice (table 6). Medical records were searched from hospitalisation (discharge letter) with additional cardiac rehabilitation or outpatient records until date of completing the survey, with exception for blood pressure measurements >140 mmHg (not at acute hospitalisation, only at follow-up).

|  | **N** | **Women, n (%)** | **Age, mean (SD)** |
| --- | --- | --- | --- |
| **Met inclusion criteria (phoned)** | **542** | **165 (30)** | **63.9 (11.0)** |
| No phone contact | 61 | 22 (36) | 61.5 (10.9) |
| No e-mail/digital illiterate (exclusion criterion) | 65 | 30 (46) | 72.1 (9.6) |
| No Dutch language (exclusion criterion) | 12 | 1 (8) | 53.6 (10.6) |
| **Total eligible** | **404** | **112 (28)** | **64.3 (10.8)** |
| Refused participation | 42 | 16 (38) | 64.7 (11.4) |
| Incomplete survey | 56 | 12 (21) | 61.9 (13.5) |
| **Completed surveys** | **306** | **84 (27)** | **63.8 (10.4)** |
| No consent for record linkage (excluded) | 43 | 14 (33) | 64.4 (10.8) |
| Incorrect timing (excluded) | 9 | 3 (33) | 63.0 (12.3) |
| **Total included** | **254** | **67 (26)** | **63.7 (10.2)** |

**Appendix table B. Patient characteristics stratified by reasons not being included in final analysis.** Response rate was calculated by dividing the total number of completed surveys (n=306) by the total number of eligible patients (N=404), so 76%. Included patients were less likely to be women compared to all patients that met inclusion criteria (26.4% vs. 30.4% women) but were similar in age (mean (SD) 63.7 (10.2) vs. 63.9 (11.0) years).

| **Variable** | **Data collection** | **Classification** |
| --- | --- | --- |
| **Dutch ethnic origin** | Country of birth was self-reported | We defined Dutch ethnic origin if participant fulfilled one of the following criteria: he or she was born in the Netherlands and at least one of his parents were born in the Netherlands; or he or she was born abroad and both parents were born in the Netherlands, based on the definition by Stronks et al.^1^ |
| **Educational level** | Highest level of education was self-reported. | Educational level was categorised according to the classification of the Dutch Central Bureau of Statistics (CBS): no or low (vocational schooling or lower secondary schooling), intermediate (intermediate vocational schooling, intermediate/higher secondary schooling) or high (higher vocational schooling or university). |

**Appendix table C. Definitions of educational level and ethnical origin.**

1. Stronks K, Kulu-Glasgow I, Agyemang C. The utility of 'country of birth' for the classification of ethnic groups in health research: the Dutch experience. *Ethn Health* 2009;**14**:255-269. doi: 10.1080/13557850802509206

2. Standaard Onderwijsindeling 2021, <https://www.cbs.nl/nl-nl/onze-diensten/methoden/classificaties/onderwijs-en-beroepen/standaard-onderwijsindeling--soi--/standaard-onderwijsindeling-2021> (cited 2021 May)
