# Supplementary material for: Patients' preferences for secondary prevention following a coronary event
Source: Prev Med Rep. 2024 Mar 8;40:102681. doi: 10.1016/j.pmedr.2024.102681 (PMC10940170; doi:10.1016/j.pmedr.2024.102681)
Supplement: Supplementary data 2 [file mmc2.docx]

1. **Wat is op u van toepassing? (Meerdere antwoorden mogelijk)**

 Roken

 Overgewicht

 Onvoldoende beweging

 Stress

 Depressie

 Hoge bloeddruk

 Hoog cholesterol

 Hoog bloedsuiker

 Geen van bovenstaande

1. **Wat vindt u op de eerste plaats belangrijk om te verbeteren? (één keuze)**

 Roken

 Overgewicht

 Onvoldoende beweging

 Stress

 Depressie

 Hoge bloeddruk

 Hoog cholesterol

 Hoog bloedsuiker

 Geen van bovenstaande

1. **Hoe gemotiveerd bent u om dit te verbeteren? (schuifbalkschaal)**
2. Niet gemotiveerd Zeer gemotiveerd (10)
3. **Hoe schat u uw kans op succes in? (schuifbalkschaal)**
4. Slechte kans op succes Goede kans op succes (10)
5. **Wat vindt u op de tweede plaats belangrijk om te verbeteren? (één andere keuze)**

 Roken

 Overgewicht

 Onvoldoende beweging

 Stress

 Depressie

 Hoge bloeddruk

 Hoog cholesterol

 Hoog bloedsuiker

 Geen van bovenstaande

1. **Hoe gemotiveerd bent u om dit te verbeteren? (schuifbalkschaal)**
2. Niet gemotiveerd Zeer gemotiveerd (10)
3. **Hoe schat u uw kans op succes in? (schuifbalkschaal)**
4. Slechte kans op succes Goede kans op succes (10)
5. **Wat vindt u op de derde plaats belangrijk om te verbeteren? (één andere keuze)**

 Roken

 Overgewicht

 Onvoldoende beweging

 Stress

 Depressie

 Hoge bloeddruk

 Hoog cholesterol

 Hoog bloedsuiker

 Geen van bovenstaande

1. **Hoe gemotiveerd bent u om dit te verbeteren? (schuifbalkschaal)**
2. Niet gemotiveerd Zeer gemotiveerd (10)
3. **Hoe schat u uw kans op succes in? (schuifbalkschaal)**
4. Slechte kans op succes Goede kans op succes (10)

Vragen over ondersteuning

1. **"Ik zou het prettig vinden wanneer ik hulp zou krijgen bij het verbeteren van (Meerdere antwoorden mogelijk):**

 Roken

 Overgewicht

 Onvoldoende beweging

 Stress

 Depressie

 Hoge bloeddruk

 Hoog cholesterol

 Hoog bloedsuiker

 Geen van bovenstaande

1. **Hoe belangrijk is het bespreken van de voor- en nadelen van een specifiek programma om uw gezondheid te verbeteren? (schuifbalkschaal)**
2. Onbelangrijk Erg belangrijk (10)
3. **"Bij het kiezen van een programma vind ik het belangrijk dat ik al mijn vragen over mijn gezondheid en de verbetering daarvan kan stellen aan bijvoorbeeld een arts, verpleegkundige, of fysiotherapeut: ..." (schuifbalkschaal)**
4. Onbelangrijk Erg belangrijk (10)
5. **Stel er is een digitale keuzehulp zoals een website of app beschikbaar die u zou kunnen helpen bij het kiezen van een programma. Zou u deze gaan gebruiken?**

 Ja

  Nee

  Weet ik niet

1. **Ik vind het aantrekkelijk om meer medicijnen te gebruiken als dit betekent dat ik verder niets aan mijn leefgewoonten hoef te veranderen.**

 Ja

  Nee

  Weet ik niet

1. **Ik ben bereid om mijn leefgewoonten aan te passen, als dit betekent dat ik minder medicijnen hoef te gebruiken.**

 Ja

  Nee

  Weet ik niet

1. **Zijn er volgens u nog andere dingen die kunnen bijdragen aan een gezonder leven voor u? Zo ja, welke? (open)**
