# Supplementary material for: Patients' preferences for secondary prevention following a coronary event
Source: Prev Med Rep. 2024 Mar 8;40:102681. doi: 10.1016/j.pmedr.2024.102681 (PMC10940170; doi:10.1016/j.pmedr.2024.102681)
Supplement: Supplementary data 3 [file mmc3.docx]

1. **What applies to you? (Multiple answers possible)**

 Smoking

 Overweight

 Insufficient physical activity

 Stress

 Depression

 High blood pressure

 High cholesterol

 High blood sugar

 None of the above

1. **What do you find most important to improve first? (one choice)**

 Smoking

 Overweight

 Insufficient physical activity

 Stress

 Depression

 High blood pressure

 High cholesterol

 High blood sugar

 None of the above

1. **How motivated are you to improve this? (slider scale)**
2. Not motivated at all Very motivated (10)
3. **How do you rate your chances of success? (slider scale)**
4. Poor chance of success Good chance of success (10)
5. **What do you find second most important to improve? (one different choice)**

 Smoking

 Overweight

 Insufficient physical activity

 Stress

 Depression

 High blood pressure

 High cholesterol

 High blood sugar

 None of the above

1. **How motivated are you to improve this? (slider scale)**
2. Not motivated at all Very motivated (10)
3. **How do you rate your chances of success? (slider scale)**
4. Poor chance of success Good chance of success (10)
5. **What do you find third most important to improve? (one different choice)**

 Smoking

 Overweight

 Insufficient physical activity

 Stress

 Depression

 High blood pressure

 High cholesterol

 High blood sugar

 None of the above

1. **How motivated are you to improve this? (slider scale)**
2. Not motivated at all Very motivated (10)
3. **How do you rate your chances of success? (slider scale)**
4. Poor chance of success Good chance of success (10)

Questions about Support

1. **"I would appreciate getting help with improving…” (Multiple answers possible):**

 Smoking

 Overweight

 Insufficient physical activity

 Stress

 Depression

 High blood pressure

 High cholesterol

 High blood sugar

 None of the above

1. **How important is it to discuss the pros and cons of a specific program to improve your health? (slider scale)**

(0) Unimportant Very important (10)

1. **"When choosing a program, I find it important that I can ask all my questions about my health and improving it to, for example, a doctor, nurse, or physical therapist: ..." (slider scale)**

(0) Unimportant Very important (10)

1. **Suppose there is a digital decision aid like a website or app available that could help you choose a program. Would you use it?**

  Yes

  No

  I don’t know

1. **I find it appealing to use more medications if it means I don't have to change my lifestyle habits.**

  Yes

  No

  I don’t know

1. **I am willing to change my lifestyle habits if it means I can use fewer medications.**

  Yes

  No

  I don’t know

1. **Do you think there are other things that could contribute to a healthier life for you? If so, what? (open)**
